# A Comparison of Times Taken for the Placement of the First Portal and Complication Rates between the Veress Needle Technique and the Modified Hasson Technique in Canine Ovariectomy Laparoscopic Surgery

**DOI:** 10.3390/ani11102936

**Published:** 2021-10-11

**Authors:** Amanda Bianchi, Francesco Collivignarelli, Massimo Vignoli, Lorenzo Scaletta, Amedeo Cuomo, Ilaria Falerno, Andrea Paolini, Roberto Tamburro

**Affiliations:** 1 Faculty of Veterinary Medicine, University of Teramo, 64100 Teramo, Italy; fcollivignarelli@unite.it (F.C.); acuomo@unite.it (A.C.); ifalerno@unite.it (I.F.); apaolini@unite.it (A.P.); rtamburro@unite.it (R.T.); 2 Veterinaria Enterprise Stp S.R.L., Via Galvani 33d, 00153 Rome, Italy; lorenzo.scaletta@veterinariaenterprise.it

**Keywords:** laparoscopy, laparoscopic surgery, Veress needle, modified Hasson technique, entry complications, first port, time of entry, laparoscopic ovariectomy

## Abstract

**Simple Summary:**

Laparoscopy is routinely used in veterinary medicine for both diagnostic and operative procedures. Laparoscopy requires the creation of a pneumoperitoneum to increase the working space, and the placement of portals to allow the insertion of instruments into the abdominal cavity. Instrument insertion is associated with most of the intraoperative complications described in the literature and can also prolong the operation time. Different techniques for reducing the complication rate and time taken for the first portal placement have been described, but there are no clear guidelines in either medical (human) or veterinary literature. In the present study, a total of 30 female dogs underwent laparoscopic ovariectomy. The times required for the first portal placement and the complication rates when using the Veress needle (10 patients) or the modified Hasson technique (20 patients) were recorded and compared. The results of this study suggest that the modified Hasson technique is faster and safer.

**Abstract:**

Minimally invasive surgery is increasingly being used in veterinary medicine. Laparoscopic procedures have several advantages compared with open surgery. These include the magnification of the field of surgery, reduced post-surgical pain and associated stress, reduced post-operative infection rates, and decreased hospitalization time. The establishment of a pneumoperitoneum is a critical step; however, this procedure can prolong the operation time, and most of the complications associated with laparoscopic surgery have been attributed to the insertion of devices into the abdominal cavity. Two main techniques have been employed to create pneumoperitoneum: the closed-entry method using the Veress needle and the open Hasson technique. The first portal is necessary to start insufflation and, subsequently, to realize the operative channel to insert the laparoscopic instruments into the abdomen. Many authors have compared the time necessary to create the first portal using different techniques in human medicine, but studies on this topic in veterinary medicine are lacking. In the veterinary medicine literature, complications associated with the creation of a pneumoperitoneum and the placement of ports include spleen, bowel, or bladder injuries; pneumothorax; and subcutaneous emphysema. The aim of the present study was to compare the times required for the placement of the first portal and the creation of pneumoperitoneum, and the rates of intraoperative complications using the Veress needle technique (VNT) and the open modified Hasson technique (MHT). The sample population comprised 30 female dogs who underwent laparoscopic ovariectomies. The dogs were randomly organized into two groups and two different entry techniques were used: Veress needle (VNT = group A) and the modified Hasson technique (MHT = group B). Complications related to abdominal entry were classified as major, in cases of organ perforation, and minor, in cases of subcutaneous emphysema and gas leakage. The VNT and MHT required 374.0 s and 242.9 s, respectively, for the placement of the first portal and for establishing pneumoperitoneum (*p* < 0.05). Their major complications rates were 20% and 0%, respectively (*p* < 0.05). Their minor complications rates were 20% and 35%, respectively (*p* < 0.05). No surgical procedures required laparotomy. The MHT was associated with a lower major complication rate and required less time to create the first portal, compared with the Veress needle technique.

## 1. Introduction

Laparoscopy is a minimally invasive surgical technique for viewing the internal structures of the abdomen [1]. Laparoscopy was initially used almost only for diagnostic procedures in veterinary medicine [2]. In recent decades, laparoscopic ovariectomy and cryptorchidectomy procedures were performed and described in the veterinary literature [3,4,5]. Currently, laparoscopic gastropexy, splenectomy, cholecystectomy, and adrenalectomy have been reported in dogs and cats [6,7,8,9]. Laparoscopic procedures have several advantages compared with open surgery: a larger field of surgery, reduced post-surgical pain and associated stress, a reduced infection rate, and less hospitalization time [10,11,12,13,14]. There is evidence in human medicine that laparoscopy is associated with a reduced overall risk of complications, compared with traditional laparotomy [2]. There are, however, risks associated with the insertion of instruments through small surgical accesses sites [15].

Laparoscopic procedures require distention of the abdomen with gas (CO_2_, air, He or N_2_O) and then inserting a laparoscope through a portal in the abdominal wall [1,16].

This is a critical step because of the potential for iatrogenic trauma to abdominal organs or vasculature during port placement [17,18]. Entry-related injuries and complications have a wide range of severities. Several studies have described possible complications associated with this step [19,20].

Two main techniques have been described to achieve the pneumoperitoneum: the closed-entry method with a Veress needle technique (VNT) and the open modified Hasson technique (MHT) [21]. The Veress needle is a laparoscopic instrument with a spring-loaded blunt stylet that is introduced blindly into the peritoneal space and then connected to the insufflation tube. Once the pneumoperitoneum is established, the first port is created [22]. The MHT provides access to the peritoneal cavity by performing a mini-laparotomy of the same diameter as the cannula, allowing the insertion of a cannula into the peritoneal cavity under direct visualization [21]. In human medicine, VN insertion may provide injury to the intra-abdominal organs (bowel, spleen, liver) vessels (aorta, vena cava, iliac vessels, mesenteric vessels), with an incidence of 0.06% to 0.5% and 0.05% to 0.3%, respectively [23]. Although vascular and visceral damage is reported with the open introduction, it is generally considered a safer method than the close technique [24].

In veterinary medicine, complications related to the pneumoperitoneum establishing included spleen, bowel, or bladder injuries; pneumothorax; and subcutaneous emphysema [25]. A fatal embolism has been reported [26]. In the veterinary literature, guidelines for establishing a safe and rapid pneumoperitoneum are lacking, and few surgical studies have been conducted [2].

In human medicine, many authors have compared the times necessary to create the first portal and pneumoperitoneum using different techniques [27,28,29], but no studies in veterinary medicine have undertaken such analyses.

The aims of the present study were to compare the times required for the placement of the first portal and the creation of pneumoperitoneum and the rates of intraoperative complications in dogs undergoing laparoscopic ovariectomy with VNT or MHT.

## 2. Materials and Methods

The inclusion criteria for this study were healthy female dogs admitted for elective laparoscopic ovariectomy. The procedures were performed by the same veterinary surgeon (LS) between July 2019 and October 2020 at the University of Teramo teaching hospital.

Dogs were randomly organized into two groups: the Veress needle technique (VNT: group A) was used for 10 dogs and the modified Hasson technique (MHT: group B) was used for the other 20 dogs.

Data collected from the medical records of dogs consisted of breed, age, body weight, preoperative systemic disease status, entry technique, the time necessary to create the first port, and number and type of intraoperative complications. In group A, the time required for first port placement was measured from skin incision, through VN insertion, to placement of the first trocar. In group B, the time required for the first port placement was measured from skin incision, through cannula positioning, to the point at which pneumoperitoneum was established.

An entry complication was defined as an injury related to initial access to the peritoneum or to the insertion of the instruments. Complications were classified as major in cases of organs perforation/trauma, hemorrhaging, and gas embolism via accidental vessel insufflation. Minor complications included subcutaneous emphysema and gas leakage.

Standard anesthetic protocols and aseptic techniques were followed for all dogs during the 30 laparoscopic ovariectomies.

### 2.1. The Veress Needle Technique (VNT) (Group A)

A 1 mm skin incision was made with a #11 scalpel blade 1 cm to the right of the umbilicus; a 150 mm long, reusable VN (Karl Storz, Tuttlingen, DE, Germany) was introduced.

The success of the VN placement was validated by introducing 5 mL of sterile saline solution through the needle (injection test). The VN was then connected to the insufflation tube and a pneumoperitoneum was established. Carbon dioxide (CO_2_) insufflation (Endoflator^®^ 50 Karl Storz endoscopy, Tuttlingen, DE, Germany) was started and continued until a pressure of 8–10 mmHg was reached.

Once pneumoperitoneum was achieved, a portal was created blindly a few centimeters caudal to the umbilicus using a 6 mm trocar cannula (Karl Storz Endoscopy, Tuttlingen, DE, Germany). A laparoscope was inserted through the portal, and visual exploration of the abdominal cavity was conducted. Any injury inflicted during the blind insertion of the needle and trocar was noted.

### 2.2. The Modified Hasson Technique (Group B)

A 5 mm skin incision was made 5 mm caudal to the umbilicus with a #11 scalpel blade. Subcutaneous fat was exposed and resected in order to visualize the linea alba. The abdominal wall was lifted using a mosquito Klemmer and incised with a #11 scalpel blade. Surgical margins were grasped with two mosquito Klemmers to lift the abdominal wall and then insert a blunt cannula (Karl Storz Endoscopy). The correct positioning of the instrument and abdominal exploration were checked through the laparoscope (Hopkins^®^ 62046 AA Karl Storz Endoscopy, Tuttlingen, DE, Germany); then, an insufflation tube was connected to the trocar. Carbon dioxide (CO_2_) insufflation (Endoflator^®^ 50 Karl Storz Endoscopy, Tuttlingen, DE, Germany) was started and continued until a pressure of 8–10 mmHg was reached.

Once the first port was created using either the VNT or MHT, a 0°, 5 mm, 29 cm laparoscope (Hopkins II, Karl Storz Endoscopy, Tuttlingen, DE, Germany) was connected to a Storz telecam SL II 202130-20 (Karl Storz Endoscopy, Tuttlingen, DE, Germany) and a Storz xenon 100 light source 201325-20 (Karl Storz Endoscopy, Tuttlingen, DE, Germany) and then introduced into the abdomen. After the visual exploration of the abdominal cavity, a second cannula (Karl Storz Endoscopy, Tuttlingen, DE, Germany) was placed cranial to the umbilicus performing a mini-laparotomy.

Laparoscopic ovariectomy procedures were performed.

### 2.3. Statistical Analysis

Statistical analysis was performed using the PRISM 9 GraphPad statistical software.

Data concerning body weight and age were recorded. The distribution was assessed by the Shapiro–Wilk test; a normal distribution was shown (*p* > 0.05). The body weights in the two groups were compared in order to obtain homogeneous groups.

The times in the two groups were then assessed using an unpaired *t*-test.

The complication rates in the VNT and MHT groups were then compared using Fisher’s exact test.

The level of significance was set at *p* < 0.05.

## 3. Results

A total of 30 female dogs met the inclusion criteria. Through a randomized selection procedure, 10 dogs were included in group A, and 20 dogs were included in group B.

The ages of the population ranged from 1 to 5 years (mean, 2 ± 1.45). The body weight ranged from 4 to 40.5 kg (mean, 19.8 ± 8.56 kg).

The mean weight for group A was 16.46 ± 6.43 kg, and that of group B was 21.34 ± 8.86 kg. The comparison of the two groups showed that the differences were not statistically significant (*p* > 0.05).

### 3.1. The Veress Needle Technique (VNT)

In Group A, the time required for the creation of a first port was 374.0 s ± 145.31 (minimum 140.0 s; maximum 650.0 s).

Major complications included splenic puncture with self-limiting hemorrhaging in 2/10 patients (20%). No procedure required conversion to open surgery. Minor complications included subcutaneous emphysema in 2/10 patients (20%).

### 3.2. The Modified Hasson Technique (MHT)

In Group B, the time required for the creation of the first port was 242.9 s ± 126.4 (minimum 60.0 s; maximum 573.0 s).

No major complications were observed, and no procedure required conversion to laparotomy. Minor complications included gas leakage from the first portal in 7/20 patients (35%).

Gas leakage was quickly managed by placing Allis forceps around the abdominal wall to limit the leak and restore the peritoneal pressure.

### 3.3. Statistical Comparison

**Figure 1:**
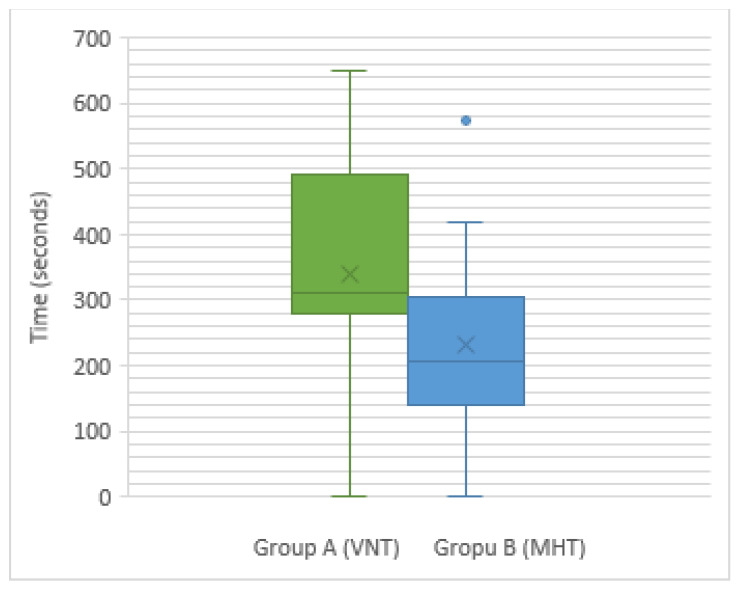
A box plot showing the distribution of times taken for the placement of the first portal and the establishment of a pneumoperitoneum in groups A (VNT) and B (MHT).

The VNT required more time to create a pneumoperitoneum compared with the MHT (Figure 1). This difference in time taken between the two techniques was statistically significant at *p* < 0.05.

VNT’s and MHT’s major complications rates were 20% and 0%, respectively. The comparison between the two groups was statistically significant at *p* < 0.05. The minor complication rates were 20% and 35%, respectively (*p* < 0.05). The comparison between the two groups was statistically significant at *p* < 0.05.

## 4. Discussion

Laparoscopy is widely used in small animal veterinary medicine for both diagnostic and therapeutic procedures [30]. Ovariectomy is the most commonly performed laparoscopic procedure in small animal veterinary practice [3,4,31,32].

Entry complications are the most widely reported complications in laparoscopic surgery [33] prompting Toro et al. to investigate two entry techniques in an attempt to reduce the complication rates [34]. They focused on the technical evolution of VNT and MHT in recent decades, and they have suggested that MHT should be preferred.

The Veress needle technique in human medicine has been associated with several major complications, such as bowel and vascular perforations [35]. In dogs and cats, both entry techniques have been used, and the risk of iatrogenic injury with either entry method has not been well determined [2].

Major complications described in veterinary medicine include gas embolisms, which have been sporadically reported [26], and bladder perforations [36]. Splenic injuries have been described in various studies; these may result in self-limiting bleeding and rarely may require conversion to laparotomy [25,37]. According to the literature, splenic lacerations are the most common complications observed during veterinary laparoscopy because of the spleen’s position immediately under the site of the first access [7]. In young dogs, generally with a very soft abdomen, the risk of iatrogenic perforation of abdominal organs or major vascular structures at the time of the introduction of the Veress needle or the first trocar cannula may increase [2]. In the authors’ experience, using the Hasson technique, lifting the abdominal wall with a mosquito Klemmer (instead of the more traditional method of using sutures) increased the space between the organs and instruments, thereby reducing the chances of potential iatrogenic organ injury.

In this study, mild and self-limiting bleeding was found in 2/30 procedures, both associated with Veress needle insertion, but no conversion to laparotomy was required. The higher complication rates associated with the VN technique are likely to be due to the blind procedure, suggesting that the surgeon’s experience may influence the complication rate [36,38]. Nylund et al., in a retrospective case series that included 141 dogs, have analyzed the rates of intraoperative complications and conversion to laparotomy during laparoscopic ovariectomy performed by veterinary students. They reported a splenic puncture in 20 patients, recorded as a minor blood loss [38].

Several complications related to the first entry have been described in the literature. Anderson and Fransson described complications related to initial access in a variety of laparoscopic procedures performed in dogs and cats: the Hasson technique was associated with only minor complications, whereas Veress needle insertion was associated with splenic injury in 2% of patients [19]. In the previous study, procedures were performed by a group of surgeons, and the surgical intervention types were extremely varied. In our study, despite the limited number of patients included in the sample, only dogs were included, and each was subjected to laparoscopic ovariectomy by the same surgeon.

VNT can also be associated with other types of complications, most likely related to the VN insertion being in close proximity to the costal arch during the inspiration phase [39]. Intercostal VN insertion has been proposed. In an ex vivo study, some authors evaluated 90 VN insertions in 15 cadavers. They observed the penetration of intra-abdominal structures or organs 47 (52%) times: 26 (55%) occurred on the left side and 21 (45%) on the right side. The liver (18%), omentum (13%), spleen (10%), diaphragm (6%), intestines (4%), and the falciform ligament (once) were the organs that were damaged [40]. The insertion of the VN through the ninth intercostal space is recommended in order to minimize its penetration into the abdomen [41]. No bladder perforation or air embolism was observed in this study even though these complications were reported in the literature [26,38].

Gas leakage is a disadvantage associated with MHT and is related to wide surgical wounds, compared with the cannula diameter. This complication is time consuming to deal with because it necessitates the interruption of the surgical procedure to restore the intra-abdominal pressure [14].

A recent veterinary medicine study described minor complications such as gas leaks as having an incidence rate of 11% [11]. In this study, the incidence was higher than this rate, probably because we used a trocar with a smooth cannula [14]. Gas leakage was quickly managed by placing Allis forceps around the abdominal wall to limit the leak and restore the peritoneal pressure. Subcutaneous emphysema is a reported complication in laparoscopic surgery [36,42]. The emphysema resulted from tissue plane disruption and subsequent leakage of CO_2_ from within the peritoneum during the surgical procedure [42] or when the Veress needle is not sharp and does not penetrate the peritoneum [2]. In this study, subcutaneous emphysema occurred in two patients of the VN group; the emphysema was self-limiting, and it completely resolved within 6 h.

In this population, MHT was associated with lower major risks, compared with the use of VNT.

The amounts of time necessary for the creation of the first port were 374.0 s and 242.9 s for VNT and MHT, respectively. No other study in veterinary medicine has quantified entry speeds or compared the rapidity of different techniques.

In the literature on human medicine, some authors have compared different techniques to identify the one that is most rapid in terms of entry. Generally, the Hasson technique is considered to be the quickest [27,28,29].

In accordance with the studies mentioned above, we found the MHT to be faster than the VN technique, and these results are statistically significant.

The main limitation of this study was the small number of patients included in the sample. A study using a larger sample would likely provide more information.

## 5. Conclusions

In conclusion, both VN insertion and HT may be considered suitable laparoscopic approaches for the establishment of a pneumoperitoneum. According to our data, MHT was faster and safer than the VNT for the creation of the first portal in laparoscopic surgery.

## Data Availability

Data are contained within the article.
